# Regeneration of cofactor NAD(P)^+^ with NAD(P)H oxidase for the production of value-added chemicals

**DOI:** 10.3389/fbioe.2025.1650600

**Published:** 2025-09-01

**Authors:** Li-Jian Zhou, Shuai-Xi Long, Tong Huan, Yue Wu, Ye-Wang Zhang

**Affiliations:** ^1^ The People’s Hospital of Danyang, Affiliated Danyang Hospital of Nantong University, Zhenjiang, China; ^2^ School of Medicine, Nantong University, Nantong, China; ^3^ School of Pharmacy, Jiangsu University, Zhenjiang, China

**Keywords:** NADH oxidase, cofactor regeneration, biocatalysis, cascade enzymatic reactions, pharmaceuticals

## Abstract

Nicotinamide adenine dinucleotide (phosphate) oxidases are the enzymes that catalyze the oxidation of NAD(P)H to produce NAD(P)^+^, which is the cofactor of many dehydrogenases. To reduce costs, cofactor regeneration of NAD(P)+ is essential for both enzymatic and whole-cell biotransformations. In the present review, the enzymatic or microbial production of rare sugars like L-tagatose, L-xylulose, L-gulose, and L-sorbose with cofactor regeneration was summarized. And the cofactor regeneration in some value-added chemicals, including acetoin, 1,3-dihydroacetone, vanillic acid, chlorolactone, acetophenone, and kinetic resolution of racemic phenylethanol by employing NADH/NADPH oxidase was also reviewed. The engineering of these enzymes by modifying the enzyme surface, reshaping the catalytic pocket, and mutating the substrate-binding domain of NADH oxidase to improve the catalytic performance for potential industrial applications was discussed in the future outlook.

## 1 Introduction

Nicotinamide adenine dinucleotide (NAD), as the most important coenzyme, could help other NAD-dependent enzymes to catalyze chemical reactions by switching between the reduced and oxidized form (NAD^+^/NADH). In eukaryotic cells, the energy could be produced using NADH in cellular metabolism. The process from NADH to NAD^+^ is generally catalyzed by NADH oxidase (NOX) and produces reactive oxygen species (ROS) in eukaryotic cells, and another main source of ROS *in vivo* is NADPH oxidase. These two categories of enzymes catalyze the NAD(P)H to produce NAD(P)^+^ for coupling with the dehydrogenases catalyzed oxidoreduction reactions for the production of enantiopure chemicals or pharmaceuticals. The increasing attention on these enzymes is due to their potential capacity to regenerate the expensive cofactor NAD(P)^+^ in industrial enzymatic preparation of value-added products with NAD(P)^+^-dependent dehydrogenases.

Most NOXs have a highly conserved catalytic residue -cysteine, located in the active site. They catalyze the oxidation reactions of NADH to NAD^+^ coupled with the four-electron transfer mode by consumption of O_2_ or the two-electron transfer mode by reducing H_2_O_2_ (Li et al., 2018). Compared with the H_2_O_2_-forming NOX, the H_2_O-forming NOX is more interesting because of its good compatibility in enzymatic reactions in aqueous solution. Similarly, the oxidized NADP^+^ could also be recycled by NADPH oxidase and follows a similar reaction mechanism.

Oxidoreductase is the largest class of enzymes, and NAD(P)^+^ is the most common cofactor of the oxidoreductase. Now, the chemical synthesis route in chemical, paper, cosmetic, and other industries is undergoing a change to biocatalytic, especially enzymatic methods. In this review, we summarized the potential industrial production of value-added chemicals with cofactor regeneration of NAD^+^/NADP^+^. In the enzymatic reactions by using coupled or combined enzymes, the strategy of protein engineering for remodeling the enzyme is also included to improve the catalytic efficiency.

## 2 Enzymatic production of rare sugars with dehydrogenase and NOX

Rare sugars are highly valued mono- or disaccharides with beneficial health effects. However, most synthetic chemical routes are both limited and economically unfeasible due to the expensive raw materials, harsh reaction conditions, and severe environmental pollution. Enzymatic transformation has become a powerful tool in the field. Many NAD-dependent enzymes are hydrogenases, which oxidize alcohols or sugars (Stolarczyk et al., 2020) to produce rare sugars. The cofactors are bound weakly to the enzyme and could be easily released from the protein. Thus, the regeneration of the cofactors is essential for the combined or cascade enzymatic reactions, which will significantly lower the cost. In the past decade, several L-form rare sugars have been successfully synthesized with dehydrogenase coupled with NAD(P)H oxidases as shown in Table 1.

**TABLE 1:** Enzymatic synthesis of the rare sugars and their applications.

Rare sugar	Enzymes	Production yield	Applications
L-tagatose	GatDH and NOX	Up to 90%	Food additive, low-calorie sweetener
L-xylulose	ArDH and NOX	Up to 93%	Pharmaceuticals
L-gulose	MDH and NOX	5.5 g/L	Anticancer drug precursor
L-sorbose	SlDH and NOX	Up to 92%	Pharmaceutical intermediate

### 2.1 Production of L-tagatose with galactitol dehydrogenase

**FIGURE 1:**
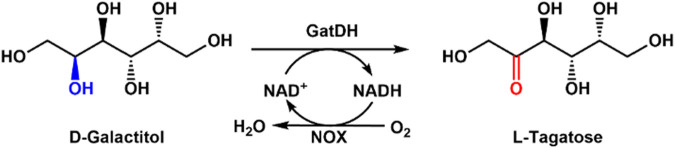
Enzymatic production of L-tagatose from D-galactitol with NOX and GatDH.

As a rare sugar, L-tagatose has great potential applications in the therapy of diabetes, food additives, and cosmetics because of its low-calorie value (Mahmood et al., 2021). Considering the increasing diabetes and obesity, low- or zero-caloric sweeteners are gaining attention in the food industry (Ravikumar et al., 2022; Ravikumar et al., 2021). The biological production method has drawn obvious achievements for L-tagatose production (Lu et al., 2024; Yuan et al., 2021). However, chemical synthesis of L-tagatose leads to low yield and by-products (Tang et al., 2022). Su et al. used galactitol dehydrogenase (*Gat*DH) and H_2_O-forming NOX (*Sm*Nox) for efficient enzymatic synthesis of L-tagatose (Su et al., 2021). The yield of 90% after 12 h reaction was obtained when the substrate concentration of 100 mM and 3 mM NAD^+^ was supplied. There was no by-product formation and a high yield of L-tagatose with low costs using this enzymatic route (Figure 1). Later, they prepared the combined cross-linked enzyme aggregates containing *Gat*DH and *Sm*Nox for the synthesis of L-tagatose (Li et al., 2022b). The combined cross-linked enzyme aggregates exhibited high thermal stability and industrial potential for the L-tagatose preparation.

### 2.2 Enzymatic production of L-xylulose with arabinitol dehydrogenase

L-Xylulose, as another important rare sugar, is usually used as an anticancer and cardioprotective agent or as a precursor for the preparation of antiviral drugs. The chemical synthesis of the L-xylulose method has disadvantages of low yield, environmental pollution, and low purity because it is difficult to separate from other isomers. The substrate for the biological preparation of L-xylulose could be L-arabinitol or xylitol.

Gao et al. reported the biotransformation of L-arabinitol to L-xylulose with immobilized whole *Escherichia coli* cells expressed L-arabinitol dehydrogenase and NOX (Gao et al., 2015). By coupling two enzymes in the cells, a molar conversion of 96% from L-arabinitol to L-xylulose was achieved. It was reported later that a product titer of 48.45 g/L for L-xylulose was reached by co-expressing the two enzymes in *E. coli* with pETDuet-xdh-smnox (Tesfay et al., 2021). They immobilized the *E. coli* cells expressing the two enzymes with entrapment, and a yield of 96% was obtained when the substrate concentration was 150 mM (Gao et al., 2016a). The group then developed a cell-free system containing the two enzymes for the synthesis of L-xylulose from arabinitol, and a final yield of 78.4% was achieved when the substrate concentration was 250 mM (Gao et al., 2016b). Then they immobilized the two enzymes onto inorganic hybrid nanoflowers to enhance the L-xylulose production, and a maximum yield of 91% was observed, which was 2.9-fold higher than the free enzymes (Patel et al., 2017). They demonstrated a sequential co-immobilization of L-arabinitol dehydrogenase and NADH oxidase for the conversion of L-arabinitol to L-xylulose recently (Patel et al., 2024). The results indicated that the co-immobilized enzymes exhibited 6.5-fold higher activity than that of free enzymes, and the maximum conversion of 93.6% was obtained.

**FIGURE 2:**
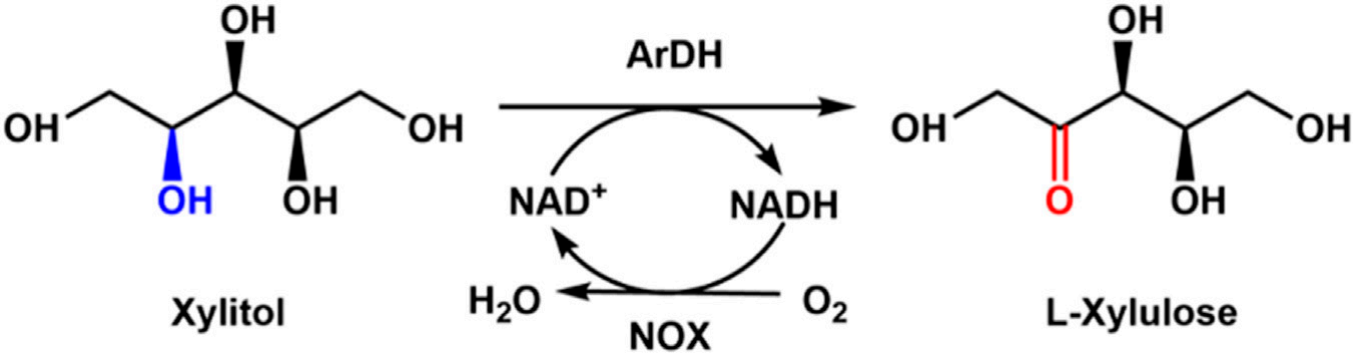
Enzymatic preparation of L-xylulose with ArDH coupled with NOX.

Generally, xylitol could be enzymatically converted into xylulose with xylitol dehydrogenase. Another possible enzymatic route is using arabinitol dehydrogenase, which is an NAD-dependent short-chain dehydrogenase that can convert xylitol to L-xylulose. Zhu et al. (2022) cloned arabinitol dehydrogenase (ArDH) from *Aspergillus nidulans* for the preparation of L-xylulose from xylitol and coupled NOX for the cofactor NAD^+^ regeneration (Figure 2). It was found that the high substrate concentration of xylitol inhibits the enzymatic reaction. A yield of 92.7% was achieved when the substrate concentration was 10 mM, but the conversion was only 18.4% when the substrate concentration was 80 mM.

### 2.3 Enzymatic synthesis of L-gulose with mannitol dehydrogenase

**FIGURE 3:**
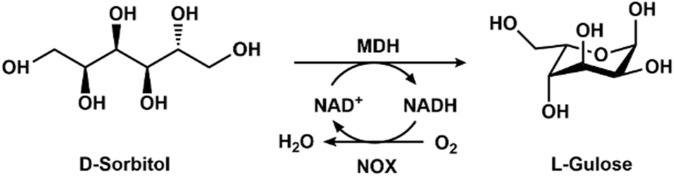
Enzymatic synthesis of L-gulose with mannitol dehydrogenase (MDH) and NOX.

L-Gulose is a building block for the preparation of the anticancer drug bleomycin and antiviral agents. The chemical synthesis routes require complex protections and deprotections of the hydroxyl groups, resulting in inconvenience, high cost, and inefficiency. Woodyer et al. constructed recombinant *Escherichia coli* harboring mannitol dehydrogenase for the production of L-gulose and L-tagatose (Woodyer et al., 2010). The productivities were only 4.6 and 0.9 g/L for L-tagatose and L-gulose, respectively. Later, mannitol dehydrogenase and NADH oxidase were co-expressed in *E. coli* by using the pACYDuet-1 vector, and the whole cell expressing mannitol dehydrogenase and NADH oxidase was employed to convert D-sorbitol to L-gulose (Figure 3). With the highly efficient cofactor regeneration, a volumetric product titer of 5.5 g/L for L-gulose was obtained after the optimization of the reaction conditions, including pH, temperature, substrate concentration, and metal ions.

### 2.4 Production of L-sorbose with sorbitol dehydrogenase

**FIGURE 4:**
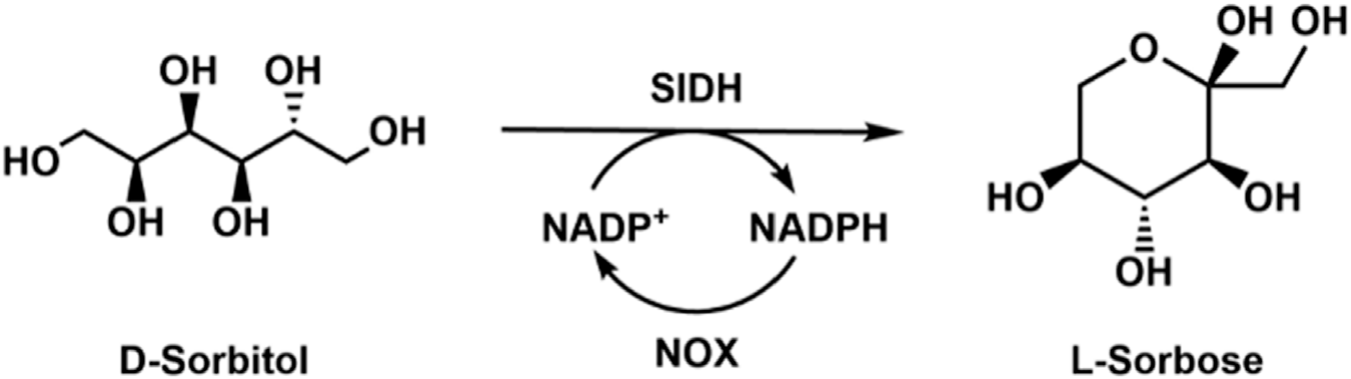
Enzymatic conversion of D-sorbitol to produce L-sorbose with sorbitol dehydrogenase (SlDH) and NOX.

L-Sorbose is an intermediate for the synthesis of L-ascorbic acid, and could be used as the starting material to synthesize the glycosidase inhibitor 1-deoxygalactonojirim and other rare sugars like L--tagatose and L-iditol. Kim et al. (2016) cloned, characterized, and immobilized a highly efficient sorbitol dehydrogenase from *Gluconobacter oxydans* G624, which showed higher activity toward D-sorbitol. However, the sorbitol dehydrogenase was inhibited by NADPH; thus, it is necessary to lower the cofactor concentration in the reaction system. They then co-expressed sorbitol dehydrogenase and NADPH oxidase in *E. coli* for the production of L-sorbose (Kim et al., 2019). After optimization of the reaction conditions, a high yield of 92% was achieved by using the whole-cell catalysts (Figure 4). The L-sorbose production was 4.1 g/L, which was 20.5-fold higher than that of *E. coli* without NADPH oxidase for the cofactor regeneration. Later, they discovered a novel NADPH oxidase which could oxidize both NADH and NADPH (Gao et al., 2019), and the L-sorbose production could be improved 53-fold with this enzyme. One drawback of the sorbitol dehydrogenase is that the working pH range (9.0–10.5) is much higher than the optimal pH of NADPH oxidase.

## 3 Enzymatic production of ketone with dehydrogenase coupled cofactor regeneration

Alcohol dehydrogenase (ADH) is the main enzyme catalyzing the oxidation of alcohol substrate to ketone product. The typical dehydrogenation reaction involves the oxidation of alcohol with the oxidized NAD(P)^+^ as the electron acceptor. A practical challenge in applying the ADHs for the oxidation of alcohol is the consumption of NAD(P)^+^. Therefore, it is feasible to recycle the cofactor NAD(P)^+^ in the ADH-catalyzed oxidation of alcohol to the synthesis of ketone.

### 3.1 Enzymatic synthesis of acetoin with acetaldehyde lyase (FLS)

**FIGURE 5:**
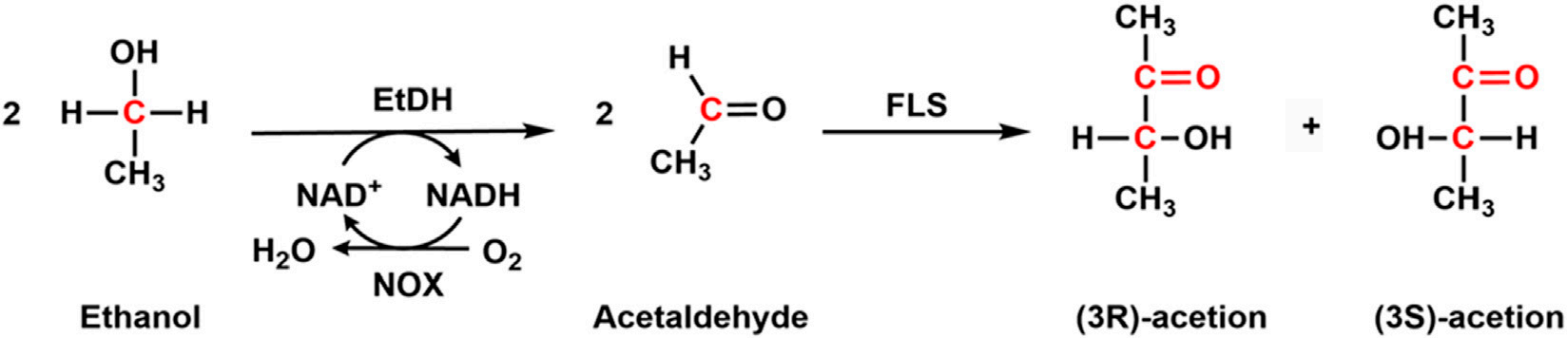
Cascade enzymatic synthesis of acetoin from ethanol with ethanol dehydrogenase (EtDH), NOX, and Formolase (FLS).

Acetoin has been used in the food and pharmaceutical industries as a flavor enhancer and precursor for alkaloid ligustrazine (Peng et al., 2020). It could be produced with chemical reduction of diacetyl, fermentation from microbial cells, or enzymatic production from ethanol (Li et al., 2022a). Although some strains, including *Enterobacter, Klebsiella, Serratia, Saccharomyces cerevisiae,* and *Bacillus* could be utilized for the production of acetoin (Li et al., 2022a), the yield was low because acetoin is an intermediate in the 2,3-butanediol metabolic pathway. By deleting the butanediol dehydrogenase encoding gene, which converts acetoin to 2,3-butanediol, and overexpressing NADH oxidase (NOX) to enhance the cofactor regeneration, the final yield of 100.1 g/L was achieved (Bae et al., 2016). Later, Guo et al. co-expressed butanediol dehydrogenase, hemoglobin, and NADH oxidase in *E. coli* for the production of acetoin, and a product titer of 86.74 g/L was obtained. To overcome the complex regulation of cell growth and purification of acetoin from the fermentation medium, Li et al. (2022a) developed an enzymatic cascade reaction for the production of acetoin. They immobilized ethanol dehydrogenase (EtDH), NOX, and formolase (FLS) onto epoxy-modified magnetic nanoparticles for the synthesis of acetoin (Figure 5). In the cascade enzymatic reactions, EtDH catalyzes the conversion of ethanol to acetaldehyde, which is subsequently transferred to acetoin with the catalysis of formolase. NADH oxidase is responsible for the regeneration of NAD^+^ in the first enzymatic step. A high conversion of 90% was achieved by using the three immobilized enzymes for one-pot biosynthesis of acetoin, suggesting that this is a promising way to produce value-added acetoin from ethanol.

### 3.2 Synthesis of 1,3-dihydroacetone from glycerol with glycerol dehydrogenase

**FIGURE 6:**
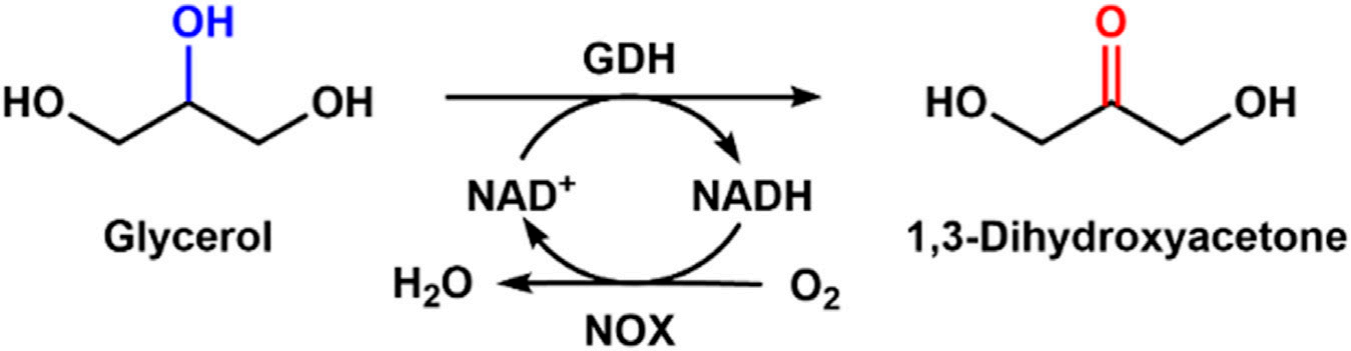
Bioconversion of glycerol with GDH and NOX mixed with NAD^+^ regeneration.

Glycerol is the byproduct of biodiesel and a cheap raw material, and it is economical to convert it to value-added chemicals (Fokum et al., 2021; Li et al., 2023). Fan et al. immobilized whole cells harboring glycerol dehydrogenase (GDH) and NADH oxidase genes through a click reaction (Fan et al., 2021). By employing the immobilized whole cell, glycerol could be converted into 1,3-dihydroxyacetone, around 400 times higher in price. The same group immobilized GDH and Nox for bioconversion of glycerol to 1,3-dihydroxyacetone, and the final product concentration of 3.5 mM was obtained, which was four times higher than that without cofactor regeneration (Figure 6). Then they combined immobilized GDH and NOX with cross-linking to form aggregates for efficient regeneration of NAD^+^; there was about a 1.5 times improvement compared with the free enzyme system (Xu et al., 2020). By using the combined cross-linked enzyme aggregate, about 4.6% glycerol was converted into 1,3-dihydroxyacetone with a final concentration of 9.4 mM. The combined cross-linked enzyme aggregate could regenerate NADH with a high speed of a turnover number of 2137 in the production of 1,3-dihydroxyacetone.

### 3.3 Enzymatic production of statin precursor with alcohol dehydrogenase and NADPH oxidase

**FIGURE 7:**
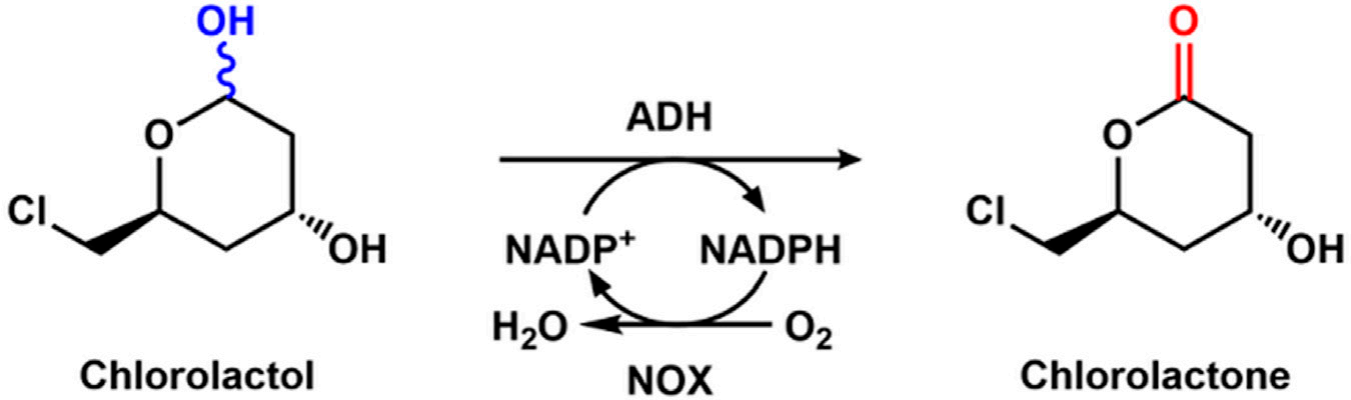
Enzymatic oxidation of chlorolactol to chlorolactone with alcohol dehydrogenase (ADH) and NOX.

Generally, an excess of reactive oxygen species will lead to plenty of devastating diseases (Panday et al., 2015). Among these diseases, cardiovascular diseases are the main leading cause of death worldwide. The treatments of the diseases are focused on using drugs such as statins to decrease low-density lipoprotein cholesterol. One attractive approach for the synthesis of statins is the enzymatic route, in which the oxidation of chlorolactol to chlorolactone is the key step. The whole cell co-expressing alcohol dehydrogenase and NADPH oxidase could help to produce chlorolactone (Figure 7), which was performed by co-expressing the two enzymes by Jiao et al. (2016). Another group immobilized alcohol dehydrogenase and NADPH oxidase onto Eupergit CM, Amino-agarose, and epoxy agarose-UAB, and used the immobilized enzymes to catalyze the conversion of chlorolactol, and a yield of 94.7% for chlorolactone was obtained (Garcia-Bofill et al., 2021).

### 3.4 Biosynthesis of 2,5-dimethylpyrazine with L-threonine dehydrogenase

**FIGURE 8:**
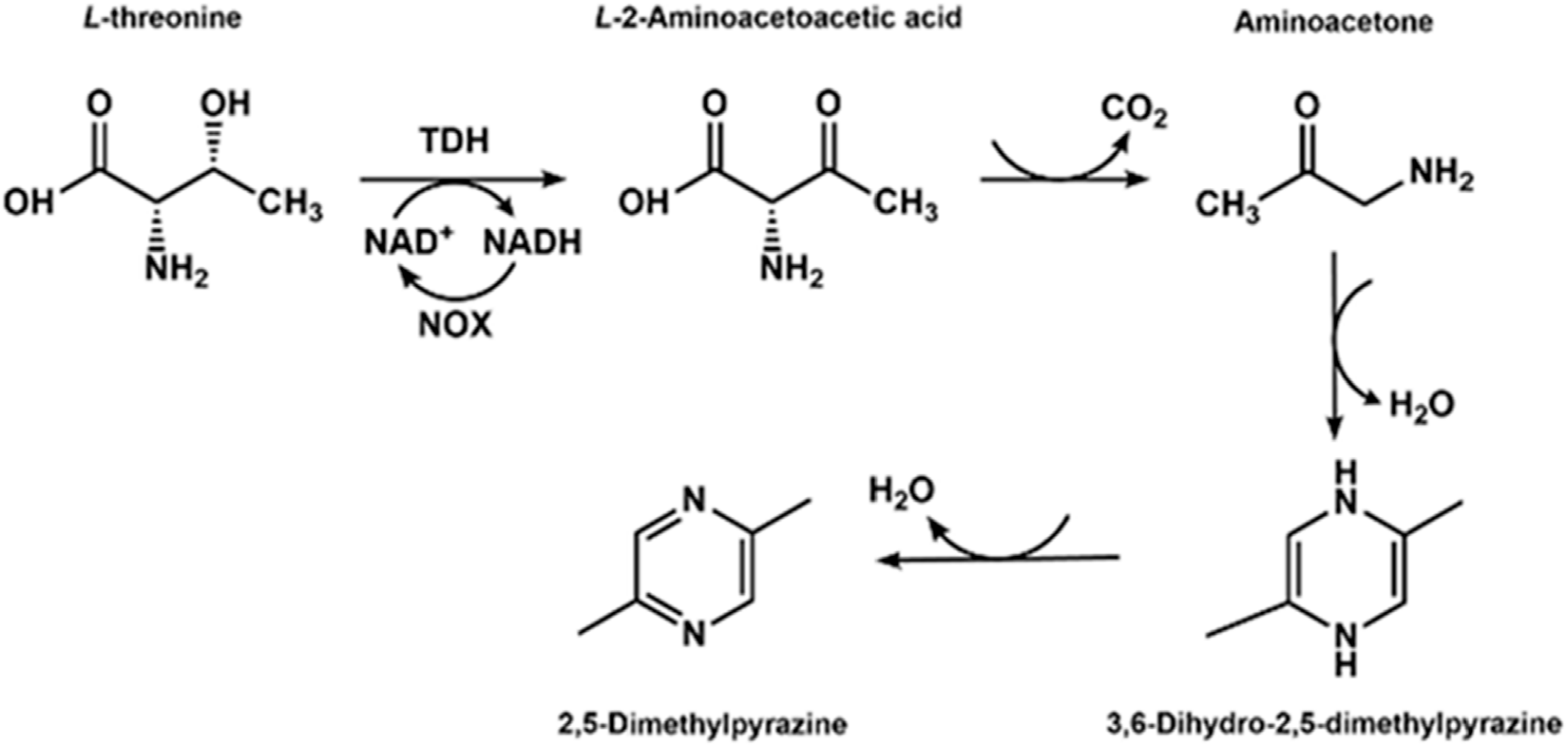
Synthesis of 2,5-dimethylpyrazine using L-threonine as a substrate with TDH.

L-Threonine is widely distributed in nature and could be prepared through microbial fermentation, making it a relatively cheap raw material. Thus, the conversion of L-threonine to a more valuable chemical is attractive and engaging for the chemical or pharmaceutical industry. Liu et al. (2023) reconfigured the L-threonine metabolic pathway in *Escherichia coli* by overexpressing NADH oxidase and L-threonine dehydrogenase (TDH) using the pACYCDuet-1 vector. The whole cell can effectively catalyze L-threonine to L-2-aminoacetoacetic acid, which could be further converted into 2,5-dimethylpyrazine. And the recombinant *E. coli* overexpressing the two enzymes can convert L-threonine to produce 2,5-dimethylpyrazine (Figure 8), which is a flavor compound and the precursor for some drugs, and a yield of 2.0 g/L was obtained with a molar conversion of 22.2%.

## 4 Enzymatic synthesis of vanillic acid with vanillin dehydrogenase

**FIGURE 9:**
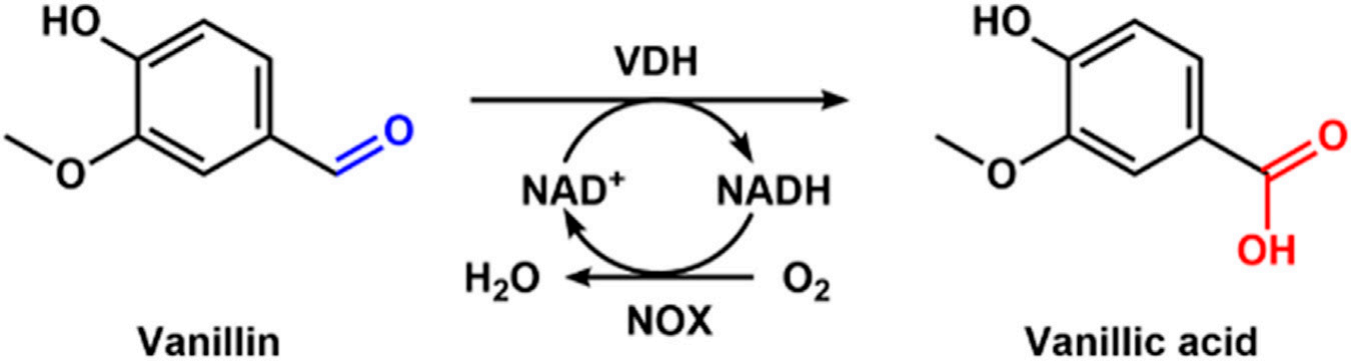
Enzymatic synthesis of vanillic acid with VDH and NOX.

Vanillic acid has been widely used in the pharmaceutical, food, cosmetic, and flavor industries because of its biological activities (Liaqat et al., 2023). Chemical synthesis of vanillic acid has some disadvantages, including low yield and environmental pollution (Ji et al., 2024). The alternative route for vanillic acid is the enzymatic transformation of lignin, which is a greener production method. Xu et al. (2024) cloned and expressed vanillin dehydrogenase (VDH) from *Bacillus ligniniphilus* L1 and NADH oxidase (NOX) from *Streptococcus pyogenes* for the biotransformation of vanillin into vanillic acid. They immobilized VDH and NOX to form cross-linked enzyme aggregates (Combi-CLVNAs). The Combi-CLVAs were used to produce vanillic acid (Figure 9). With fed-batch addition of vanillin to the reaction mixture, the production of 44.21 mM vanillic acid was obtained, which was 22.5-fold higher than without NOX in the presence of 2 mM NAD^+^. After 4 cycles, a total of 149.98 mM vanillic acid was harvested using Combi-CLVNAs. Finally, 38.69 mM vanillic acid was prepared from vanillin-rich extracts derived from lignin depolymerization with combi-CLVNAs. In the whole process using combi-CLVNAs, there were no by-products, which provides a green way for vanillic acid production.

## 5 Kinetic resolution of racemic phenylethanol with alcohol dehydrogenase

Optically active alcohols are important pharmaceutical or chemical intermediates and could be biosynthesized with alcohol dehydrogenase. However, these enzymes are highly dependent on the oxidized NAD cofactors, which are too expensive to be used stoichiometrically. So, scientists tried to use NADH oxidase to regenerate the cofactor. (*R*)-alcohol dehydrogenase from *Lactobacillus brevis* (*Lb*ADH) was used to enantioselectively oxidize racemic phenylethanol to produce acetophenone, and another product is (S)-phenylethanol, and NADH oxidase from *Lactobacillus sanfranciscensis* was employed to reduce O_2_ to H_2_O, coupling with the regeneration of cofactor NAD^+^ (Riebel et al., 2003). The mutant of *Lb*ADH G37D prefers NAD^+^ as the cofactor and is coupled with LsNox to yield 50% conversion of racemic phenylethanol to (*S*)-phenylethanol and acetophenone. It was found that more than 100 turnovers were obtained depending on the relative concentration of alcohol to cofactor. The regeneration system can also recycle NADP^+^ with the same NADH oxidase.

**FIGURE 10:**
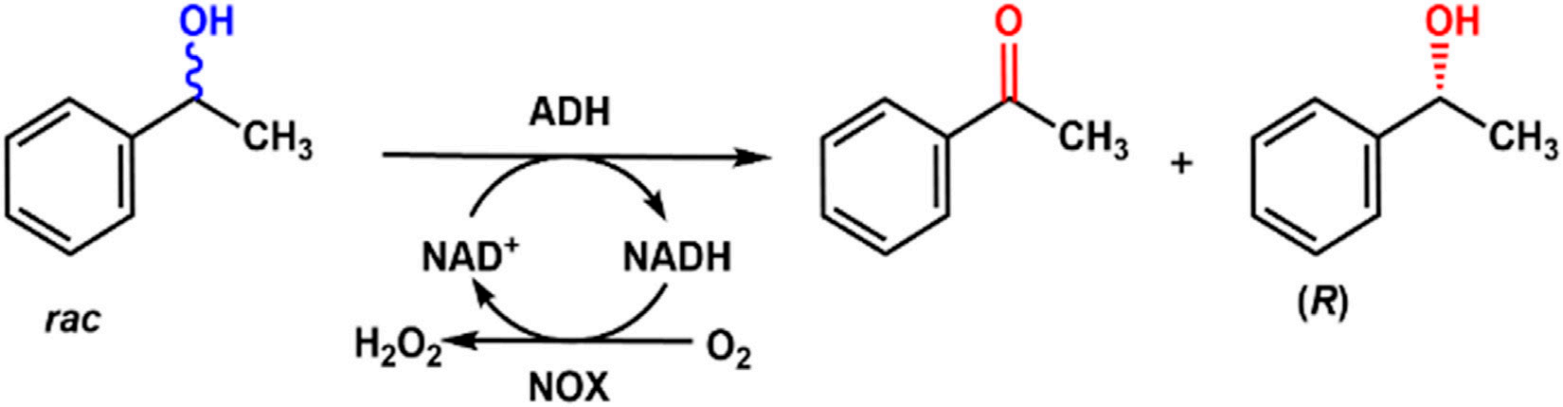
Enantioselective oxidation of racemic phenylethanol with alcohol dehydrogenase (ADH) and NOX.

Wu et al. (2022) designed and constructed fusion proteins containing alcohol dehydrogenase (ADH) and NADH oxidase for the production of chiral alcohols (Figure 10). The NOX in the fusion proteins exhibited much higher specific activities than that of the individual enzyme, which could regenerate NAD^+^ in the enantioselective oxidation of racemic 1-phenylethanol. The fusion proteins linked NOX at the N-terminus showed higher turnover numbers than the individual enzyme, especially at low cofactor concentrations.

**FIGURE 11:**
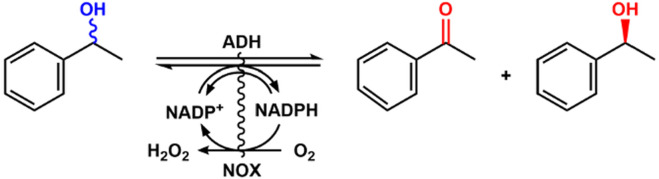
Kinetic resolution of racemic phenylethanol with fused NOX and ADH.

To expand the application of alcohol dehydrogenase for alcohol oxidation, the cofactor must be recycled to make the process economical because of the stoichiometric consumption of the expensive NADP. Aalbers and Fraaije (2019) designed an artificial alcohol oxidase by fusion of alcohol dehydrogenase and NADPH oxidase for continuous oxidation of alcohol (Figure 11). In this system, a catalytic amount of NADP^+^ was required, and the purified enzymes could perform a good catalytic efficiency of 69%–99% conversion and 99% ee with racemic phenylethanol as the substrate. Compared with other fusion proteins, the NADH oxidase fusion partner has some advantages and could be a valuable tool for the development of robust catalysts. The NADH oxidase was immobilized for the regeneration of NAD^+^ in the kinetic resolution of racemic phenylethanol (Rocha-Martín et al., 2011).

## 6 Conclusion and future outlook

Now, all the chemical synthesis and transformations are undergoing the shift from chemical methods to biological routes. With the advances of synthetic biology, important enzymes and whole cells are engineered and have great potential applications in pharmaceutical, food, cosmetic, petrochemical, and other industries. Oxidoreductases, as the largest enzyme class, have essential biological functions in life, and will have a huge future market in the industry. However, although the cofactor is expensive, most oxidoreductases need cofactors to transfer electrons, atoms, or chemical groups in a stoichiometric amount. From the industrial aspects, the regeneration of the cofactor will lower the cost and make the preparation of valuable chemicals economical. NAD(P)H oxidases have wide applications in biotechnology and are used as genetic tools to regulate physiological roles of compartment-specific changes in NAD(P)^+^/NAD(P)H equilibrium in living cells.

**FIGURE 12:**
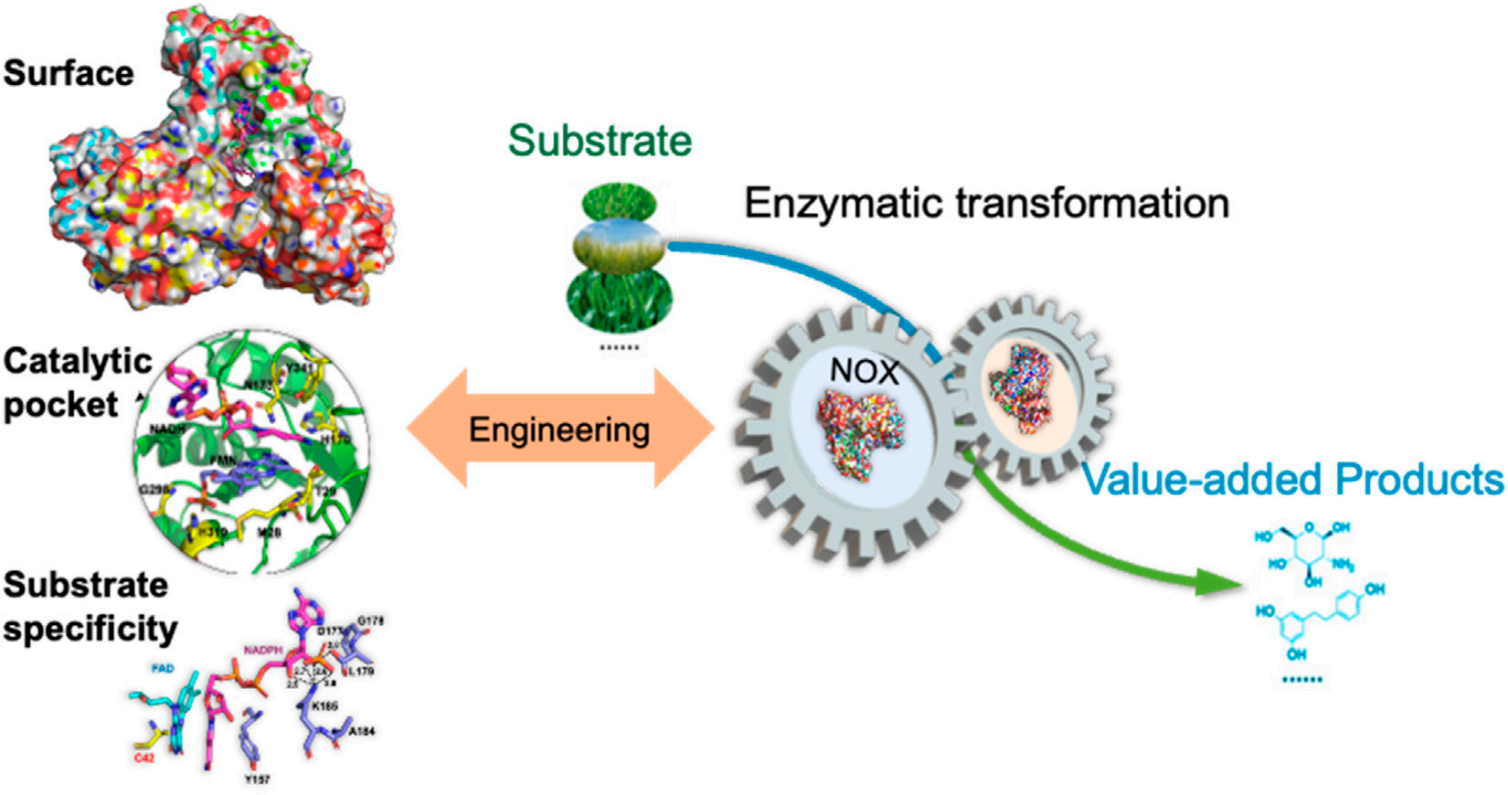
Protein engineering of the surface, catalytic pocket, and substrate binding domain to improve the catalytic activity of NOX to enhance the production of value-added chemicals.

Much research work has been done on the discovery, catalytic mechanism, biochemical characterization, and deciphering the structure of NAD(P)H oxidase. It is easy to conclude that value-added chemicals could be prepared with NAD(P)-dependent dehydrogenases, which need the cofactor regeneration with NAD(P)H oxidases. However, there are some biocompatibility issues for the enzymes. It is known that most dehydrogenases have a pH optimum around 8.0–10.0, and some NAD(P)H oxidases are not active in the pH range. For example, the optimal pH of NOX from *Lactobacillus rhamnosus* is 5.5, which cannot work together with most dehydrogenases (Zhang et al., 2012). To overcome the pH incompatibility, the enzyme was engineered to shift the pH optimum and working pH range by mutation of Asp251 to Arg, and the activity was improved 1.44-fold than the wild-type at pH 7.5 (Zhou et al., 2021). NADPH is more difficult to recycle, and the price of NADPH is higher than that of NADH; So, the NAD(P)H oxidases that could regenerate both NAD^+^ and NADP^+^ are more interesting for industrial production. Generally, engineering the surface, catalytic pocket, and the substrate binding domain is a practical approach for enhancing the production of value-added products (Figure 12). These enzymes have achieved notable enhancements in catalytic efficiency, substrate specificity, and operational stability through site-directed evolution and rational design. The dual-substrate NOXs with the ability to switch substrate from NADH to NADPH open new avenues for their industrial applications in the biosynthesis of high-value chemicals. Furthermore, the adaptation of these enzymes to varying pH and temperature conditions is crucial for their integration into industrial processes, ensuring compatibility and efficiency. Discovering new enzymes with high specific activity on both substrates would benefit the applications (Gao et al., 2019; Li et al., 2018), or engineering the enzyme protein to switch the substrate specificity by mutation of Leu179 to Ser (Li et al., 2019). There was a report that engineered NADH oxidase for the regeneration of oxidized non-natural cofactor nicotinamide cytosine dinucleotide (NCD), which could expand the development of NCD-linked redox system (Wang et al., 2025). There is no doubt that more and more NAD(P)-dependent dehydrogenases will be applied in industry with the regeneration of NAD(P)^+^ by NAD(P)H oxidase. The synergy achieved by coupling engineered NADH oxidase with other enzymes in biocatalytic systems has demonstrated remarkable improvements in product yield and reaction efficiency.

NAD(P)H oxidases catalyze the oxidation of NAD(P)H to regenerate NAD(P)^+^, in which the product is the oxidized form of the chemicals. The contrary reaction for the production of reduced forms of chemicals to form a chiral center is attracting more attention because of the sharply increasing demand for enzymatic transformations. To regenerate the reduced form NAD(P)H, many enzymes, including glucose dehydrogenase (Zhu et al., 2019), formate dehydrogenase (Wu et al., 2025), and phosphite dehydrogenase (Zhang et al., 2022) have been employed. However, to drive the reaction to the product, a second substrate and product must be introduced for the regeneration enzyme, which will complicate the separation and purification of the products. Recycling or regenerating the reduced form NAD(P)H of the cofactor without introducing by-products is a challenge for enzymologists and chemists.

## Abbreviations

ADH, alcohol dehydrogenase; ArDH, arabinitol dehydrogenase; EtDH, ethanol dehydrogenase; FLS, formolase; GDH, glycerol dehydrogenase; *Gat*DH, Galactitol dehydrogenase; MDH, mannitol dehydrogenase; *Sl*DH, sorbitol dehydrogenase; TDH-L-threonine dehydrogenase; NAD, Nicotinamide adenine dinucleotide; NOX, NADH oxidase/NADPH oxidase; ROS, reactive oxygen species; VDH, vanillin dehydrogenase.
